# *MarkIt:* A Collaborative Artificial Intelligence Annotation Platform Leveraging Blockchain For Medical Imaging Research

**DOI:** 10.30953/bhty.v4.176

**Published:** 2021-06-22

**Authors:** Jan Witowski, Jongmun Choi, Soomin Jeon, Doyun Kim, Joowon Chung, John Conklin, Maria Gabriela Figueiro Longo, Marc D. Succi, Synho Do

**Affiliations:** 1 Laboratory of Medical Imaging and Computation, Massachusetts General Hospital and Harvard Medical School, Boston, MA, USA; 2 Division of Emergency Imaging, Department of Radiology, Massachusetts General Hospital and Harvard Medical School, Boston, MA, USA; 3 Medically Engineered Solutions in Healthcare (MESH) Incubator, Massachusetts General Hospital, Boston, MA, USA

**Keywords:** artificial intelligence, data annotation, learning from crowds, blockchain, rewarding system

## Abstract

Current research on medical image processing relies heavily on the amount and quality of input data. Specifically, supervised machine learning methods require well-annotated datasets. A lack of annotation tools limits the potential to achieve high-volume processing and scaled systems with a proper reward mechanism. We developed MarkIt, a web-based tool, for collaborative annotation of medical imaging data with artificial intelligence and blockchain technologies. Our platform handles both Digital Imaging and Communications in Medicine (DICOM) and non-DICOM images, and allows users to annotate them for classification and object detection tasks in an efficient manner. MarkIt can accelerate the annotation process and keep track of user activities to calculate a fair reward. A proof-of-concept experiment was conducted with three fellowship-trained radiologists, each of whom annotated 1,000 chest X-ray studies for multi-label classification. We calculated the inter-rater agreement and estimated the value of the dataset to distribute the reward for annotators using a crypto currency. We hypothesize that MarkIt allows the typically arduous annotation task to become more efficient. In addition, MarkIt can serve as a platform to evaluate the value of data and trade the annotation results in a more scalable manner in the future. The platform is publicly available for testing on *https://markit.mgh.harvard.edu*.

As the field of supervised machine learning (ML) and artificial intelligence (AI) becomes more mature, researchers working on medical ML or AI aim to integrate more data to improve their models, as opposed to merely changes in the algorithm architecture (1–3). Ensuring high-quality annotations is critically important in medicine: imaging can be non-diagnostic, and intra- and inter-observer variability is high. About 25% of radiologists do not agree with other radiologists’ diagnoses and 30% do not agree with their own previous decisions (4). The ultimate ground truth, such as pathology reports, is not always available, and trained models often rely on the ‘soft’ annotated ground truth. Biases from poorly annotated datasets can result in critical consequences for ML algorithms in clinical use. Crowdsourcing annotations have been investigated for decades, including methods of combating noisy labels (5–9). However, to date, there have been few available collaborative annotation platforms for ML systems capable of handling medical imaging datasets.

Improving the quality of the database requires the participation of well-trained experts and a thorough curation process, which is based on voluntary commitment. It is important to consider that crowdsourcing data collection methods can be easily contaminated by mislabeling caused by undertrained participants. Consider that the value of the data or accuracy of annotation may be easily estimated. In this scenario, it is possible to construct a high-quality dataset with an appropriate proportion of positive features for AI training by exchanging or trading datasets between researchers and vendors. Furthermore, this transaction can be fairly evaluated and securely monitored. This research study introduces a web-based, zero-footprint collaborative annotation tool for medical imaging data, *MarkIt*. The proof-of-concept experiment includes implementing the platform with pre-trained AI models and blockchain features, and using them to create preliminary annotations of a chest X-ray dataset for classification tasks.

## Methods

**Fig. 1:**
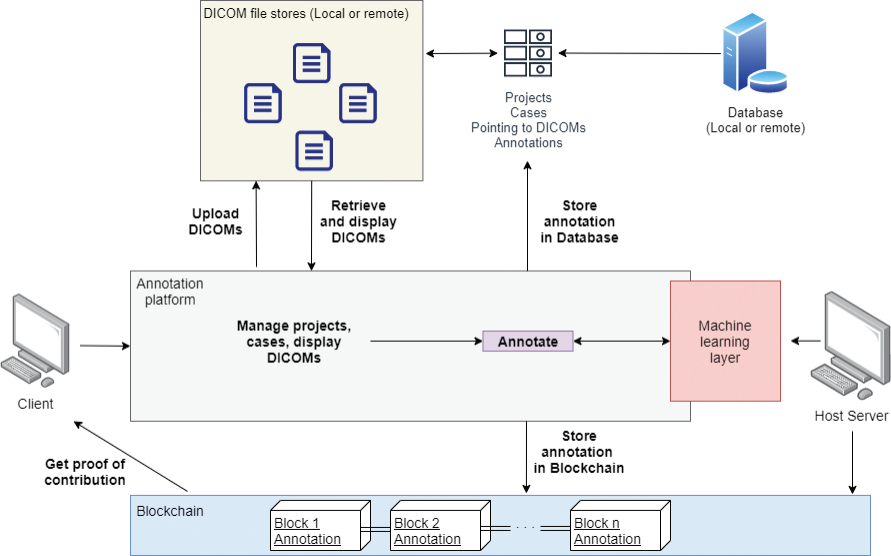
High-level data flow of *MarkIt*. Blockchain ledger storage and access are separate from the regular database. Artificial intelligence interface allows to train new models based on gathered annotations and make annotation suggestions to speed up the workflow.

The study was approved by Institutional Review Board of our hospital. The platform is currently available online using a modern web browser without the need for downloading or installing additional software. Users are required to have an internet connection and to create an account to access the platform. The platform is implemented, including several modularized functions, as shown in Fig. 1.

**Fig. 2:**
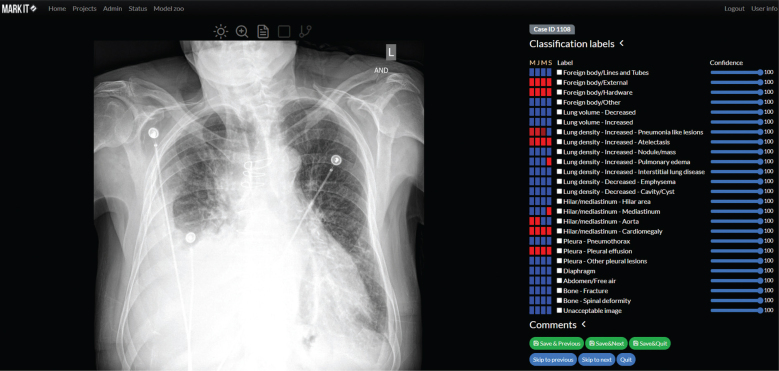
Main module for image annotation combining basic DICOM viewer features (i.e. change brightness or contrast, zooming, etc.), displaying radiological reports and annotation tools (above the X-ray image). Annotators can determine their confidence with regard to each label (blue bars on the right) and preview annotators by other team members (blue and red rectangles).

**Fig. 3:**
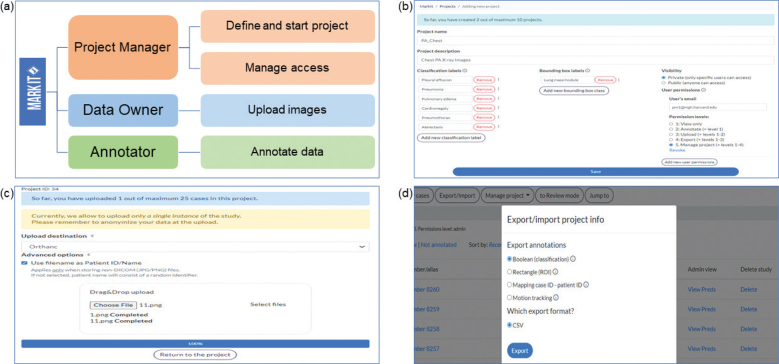
Various stakeholders and their roles in managing large projects for scalable medical image datasets. (a) The platform described in this study facilitates workflow for all parties, maximizing their focus on a single part of the process, project managers defining the project and managing access levels, data owners on image upload, and annotators on labeling. (b) Project managers can coordinate projects by specifying labels in accordance with planned AI tasks, controlling visibility for all users, as well as granting and revoking permissions for annotators (c) Data owners can upload images with additional options for choosing desired data storage systems and file naming conventions. (d) Project managers can also export project-related data, including annotations by all team members and information about the time spent on labeling by users.

The main module for image annotation consists of a simple Digital Imaging and Communications in Medicine (DICOM) viewer and labeling tools. The DICOM viewer allows annotators to change image brightness and contrast, and to zoom-in to read images in full resolution (Fig. 2). For classification, the *MarkIt* system provides the ability to save Boolean annotations and associated confidence levels as six iterative grades (0, 20, 40, 60, 80, and 100%). For object detection problems, rectangular or free-line region-of-interest (ROI) annotations are available. Users can easily define their subcategories of labels for the specific target projects when they set up a project. This can be easily modified if the user is either an owner or manager of the project (Fig. 3).

The DICOM viewer and annotation tools were implemented utilizing the open-source Cornerstone.js JavaScript library [https://docs.cornerstonejs.org/]. Cornerstone.js includes features for image loading, parsing, decoding, and tools commonly encountered in DICOM viewers.

Our platform is capable of fetching images from any vendor-neutral DICOM storage. We implemented a connection with both standard Picture Archiving and Communication System (PACS) systems and DICOM web-based RESTful web services and application programming interfaces (APIs). In this proof of concept, we utilized Orthanc [https://www.orthanc-server.com, Liege, Belgium] and Google DICOM Store (through Google Healthcare API, CA, USA), where image retrieval can be performed through WADO (Web Access to DICOM® Persistent Objects) protocols (10). Connecting to standard PACS systems and fetching images with the C-GET protocol were also implemented. Additionally, our platform allows users to use non-DICOM image files, common in large-scale non-volumetric medical datasets, for example, National Institutes of Health (NIH) and Stanford chest X-ray datasets (11, 12). In *MarkIt*, all patient information is anonymized.

All stored studies are organized privately by users into projects. The platform allows project managers to assign access privileges to the project for other readers and annotators, thereby preventing unwanted access to sensitive medical data. In addition, specific users can have various access levels, limiting some features, such as data export, progress tracking, project statistics, and management (Fig. 3). All annotations are saved in the database and include information on the time spent on a single case from the moment the image is completely loaded to the click of the submit button, the time of mouse clicking for labeling, or motionless duration to evaluate each label’s duration of tasks.

Users are allowed to export annotations in comma-separated values (CSV) format for both classification results and ROI labels. It is also possible to import radiological reports to the platform, matching them with specific cases. *MarkIt* is developed to optimize annotation workflow, especially in large-scale datasets with multiple collaborators and stakeholders, and their roles were taken into account when designing the platform, as shown in Fig. 3.

The *MarkIt* system is also connected with a locally developed AI inference RESTful (Representational Status Transfer) service, running on the same device through a Docker container [https://www.docker.com, CA, USA]. This service includes four AI classification models for chest X-ray data, predicting view position (i.e. AP: anterior-posterior vs. PA: posterior-anterior), pathological features, gender, and age. View position and gender predictions are framed as binary classification tasks, feature prediction as a multi-label classification task, and age prediction as a regression task. We plan to further expand the collection of available pre-trained models and to improve the performance of current models by changing datasets or model architectures.

Users of the platform can request model prediction on the loaded image in real-time by passing the input through a GPU-accelerated inference service. Images are sent to the service from DICOM storage via an API that evaluates sent data and returns predictions, probabilities, and feature activation maps.

Predictions are returned to users onto a *MarkIt* user interface. Feature activation maps in the form of gradient-weighted class activation mapping (Grad-CAM) (13) are overlaid over a DICOM image as a Red-Green-Blue-Alpha (RGBA) matrix with adjustable opacity.

The *MarkIt* system provides a ‘review mode’ to evaluate discrepancies between annotators. The process of annotating medical imaging data for ML purposes is different from making diagnosis in the actual clinical environment, and annotators may have different standards for determining whether a particular feature exists. Therefore, a plan to resolve disagreements is desirable to maintain consistency of the dataset. Project managers may save time by running smaller sample projects before the main annotation project to assess the presence of various problems.

This function shows the labeling results and reliability of annotators in the form of a heatmap, and allows the second annotators to check whether their results are in agreement with the preceding annotators. With this mode, annotators can develop better annotating strategies and prevent trial and error in main annotation projects.

Furthermore, we can take advantage of this mode for training or education purposes. Before implementing the main project, it has a combined function that reduces unnecessary mistakes and pre-training sessions using the review mode. Users can quickly check and resolve their discordance problem in pre-training sessions with *MarkIt* using a review mode.

Finally, the presented platform includes an experimental blockchain implementation to partially track user activity, including annotating images, and uploading and exporting data. In the future, blockchain may facilitate better security and traceability of medical imaging datasets, especially when considering global platforms that deal with sensitive data, such as medical imaging.

## Experiments and results

Three fellowship-trained radiologists classified the chest X-ray images as proof of concept in the private project. In total, 1,000 anonymized PA-view chest X-ray images with DICOM format of Massachusetts General Hospital were uploaded to the *MarkIt* platform. Twenty-five classification labels were determined and assigned to the project (Fig. 2, Supplementary Material 1). We compared these binary classification results with AI-generated prediction results from in-house data, generated the time statistics, and estimated the task difficulty. Detailed AI algorithms are not included because they are not related to the scope of this study. You can think that it is not different from the general ML or AI algorithm.

To suggest a clear analysis method, we only concentrated on seven critical labels with a clinically high value (i.e. interstitial lung disease, pneumonia, pulmonary edema, pleural effusion, cardiomegaly, pneumothorax, and atelectasis). Other features can also be analyzed in the same way and have similar characteristics.

The inter-rater agreement between the three annotators was measured (0.90) by matching the results of three annotators, and we also measured Fleiss’s Kappa value (0.63) to assess the reliability of agreement between three raters when assigning categorical ratings in this case, seven pathological feature annotations. Of 7,000 labels that were annotated, in total (7 labels times 1,000 images), 370 were labeled by all annotators as positive and 5,954 as negative.

**Fig. 4:**
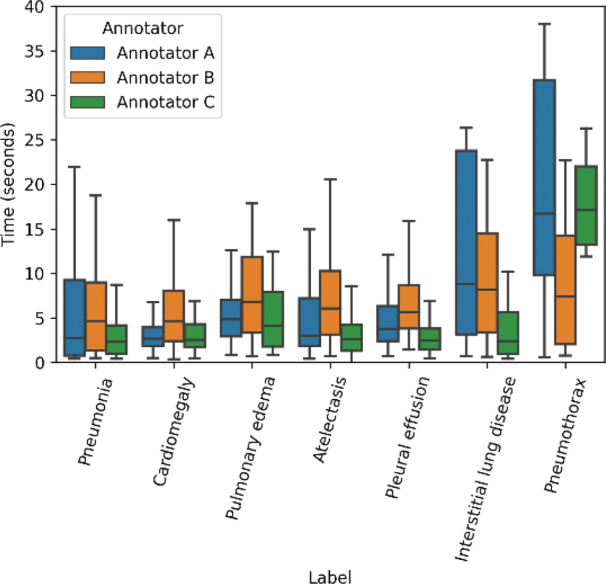
Time distribution of each label and annotators. Annotator C spent the shortest time among the annotators for all labels, except pneumothorax. The pneumothorax labeling requires the longest time, in general, likely due to the use of ancillary tools such as zoom to view the pleural line, compared with the cardiomegaly cases requiring the shortest time.

The remaining 676 labels had differences in assessments between readers. The 95% confidence interval of total mean labeling time was 6.16 ± 0.21 sec, and cardiomegaly cases took the shortest labeling time (4.63 ± 0.54 sec); in contrast, pneumothorax cases required the longest labeling time (13.92 ± 3.93 sec, Supplementary material 2). As shown in Fig. 4, distribution of the time spent annotating varied depending on both the label type and radiologist.

When annotations on a new dataset are received, it is important to understand the following: 1) how much is the data worth? 2) How much is any annotation worth? 3) Which annotator contributed and how much? For that, we formulate the value of the data based on the dataset characteristics, time cost of entering the annotation, and its annotation accuracy. The average labeling time was identified as an indicator for estimating the labor in the annotation. To calculate accuracy, we measured agreement between the annotated label and pseudo-ground truth, defined as the majority rule between annotators.

**Fig. 5:**
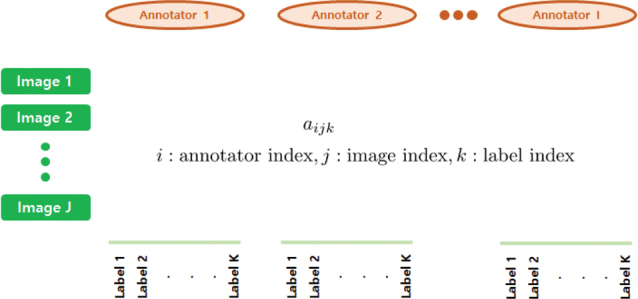
Annotation evaluation sheet.

To evaluate the annotator’s contribution for CXR PA dataset, we exported the binary classification data and generated the annotation evaluation sheet consisting of True or False. As shown in Fig. 5, for the k-th label of j-th image, the i-th annotator’s annotation results (True or False) are recorded as *a_ijk_*. We consider the seven labels (Fig. 4) for 1000 cases annotated by three annotators, so I=3, J=1,000, K=7. Using the table, we devise an Algorithm 1 to estimate each annotators’ contribution, level of challenge of each image and task, respectively, and to evaluate each annotator’s reward (Eq. 1).

In Algorithm 1, we evaluate the contribution credit of i-th annotator for the k-th label of j-th image *r_kji_*, the value of k-th label of j-th image for data *D_kj_*, and the task value *T_k_*. Basically, the lower the correct answer rate, the higher the value of the image and task and the annotators’ contribution. We set the pseudo answer *a_kj_* for the k-th label of j-th image and consider it as a ground truth for each task (k-th label of j-th image). To count the laboring factor, we put the normalized time mean for k-th label *t_k_*. Here, *c_{condition}_* is a characteristic function having the value 1 if the condition is true, otherwise 0.

**Algorithm 1:**
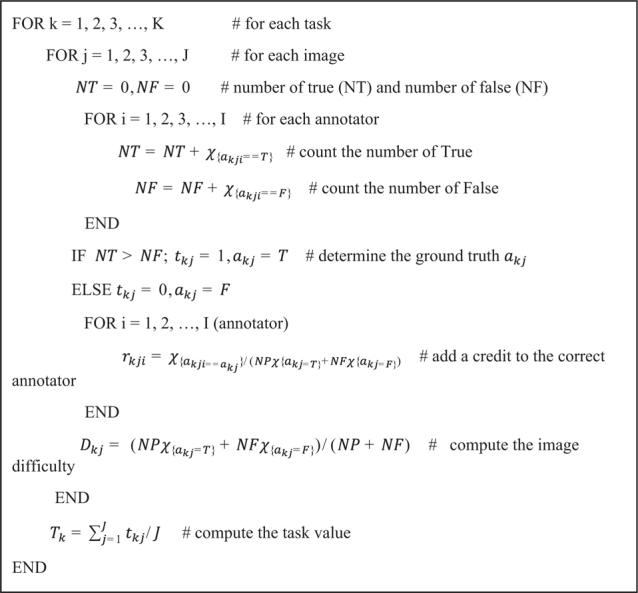
An algorithm for calculating reward factors.

It is obvious that *x* ∈ [*a,b*] can be normalized oppositely (b maps to −1, a maps to 1) as $\frac{2}{b-a}\left(\frac{a+b}{2}-x\right)\in \left[-1,1\right]$ Applying it on *D_kj_* ∈ [0,1] and *r_kji_* ∈ [0,0.5], we scale them as follows:


$${\tilde{D}}_{kj}=2*\left(0.5-D_{kj}\right)\in \left[-1,1\right]$$

$${\tilde{r}}_{kji}=4*\left(0.25-r_{kij}\right)\in [-1,1].$$

Each annotator’s reward *reward* (*i*) can be formulated as a linear combination of each factor as follows:

$$reward\;\left(i\right)=\left(Total\;Budget\right)*\sum_{k=1}^{K}\left(T_{k}+{\bar{t}}_{k}\right)\sum_{j=1}^{j}{\bar{D}}_{kj}{\tilde{r}}_{kji}/\sum_{i=1}^{I}\sum_{k=1}^{K}\left(T_{k}+{\bar{t}}_{k}\right)\sum_{j=1}^{J}{\bar{D}}_{kj}{\tilde{r}}_{kji}$$ (Eq. 1) .

This approach considers the information on the dataset and the estimated annotation quality and the time it takes to determine it, and the label-specific accuracy of the annotator.

For our experiment, we assumed the value of the entire dataset as 1000T MED Token (a cryptocurrency used in the current research; any cryptocurrency could potentially be used in the platform) for the seed money of the data trading system, and distributed the *MarkIt* currency to three annotators (i.e. Annotator A: 371T, Annotator B: 347T, and Annotator C: 282T MED Token) from (Eq. 1, Supplementary Material 3).

Equation 1 is the most fundamental formula that can be modified in different ways depending on the situation, for example, the importance of various factors can be considered through the sum of weights. In reality, the value of data will change. Initially, we will start with a small amount of data, and the performance of AI developed using these data will also have limitations.

As a large amount of data are, however, added gradually and more annotators label the data, the data’s value will increase, and the entire data’s value will increase. In this case, what is calculated by Equation 1 is repeated according to the change in the quantity and quality of the data, and the value will change accordingly.

To prevent non-expert annotators from exceeding experts in number making wrong ground truth, we introduced the AI as a quality controller. Another research group developed this AI in our laboratory for chest X-ray analysis. According to the AI result, we set a temporary ground truth and assumed that it has better performance than a random choice (i.e. coin tossing). We calculated Cohen’s kappa values between AI and each annotator. If this value was greater than 0.05, we assumed that this annotator has a better prediction power than random selection. All annotators show better performance than the threshold in each label (Supplementary Material 4), so we used all labels for reward calculations [https://github.com/MGH-LMIC/annotation_blockchain_share_calculation].

We tested a Panacea blockchain in our implementation, which is developed on top of Cosmos SDK [https://cosmos.network/sdk] and Tendermint framework [https://tendermint.com/sdk/]; however, *MarkIt* can be integrated into any framework that supports blockchain implementations. Interacting with the blockchain can be executed through the RESTful API and the command-line interface (CLI) for a Go (programming language) application. In our experiments, we were saving user activity as hashed information in separate transactions on the blockchain. Using this information, we tried to calculate the dataset’s value and estimated the awards for annotators via a blockchain currency to facilitate accurate annotation. In addition, you will be able to add various applications and services. However, in this proof-of-concept experiment, everything is not completely implemented and is continuously added in the future.

## Discussion

Crowdsourcing annotations for training AI models have been used extensively and effectively for various computer vision tasks. However, in the medical imaging field, annotation tasks require the expertise of a trained radiologist, and even for simple tasks, crowdsourced annotations can be noisy or inaccurate. A successful crowdsourcing platform include the following benefits:

- faster production of high-quality labeled datasets,
- more economical cost of obtaining annotations on the large datasets,
- accelerated development of ML or AI for multiple medical imaging tasks.

The presented platform allows researchers and commercial vendors to accelerate the annotation and development of medical imaging datasets. Current tools enable labeling for classification and object detection tasks, and provide various data and project management tools. Integration of AI can already assist users by providing activation maps as a suggestion of the area of interest. In the future, connected ML or AI models will further speed up the annotation process by offering users AI-processed labels.

This study confirms that quick annotation of large-scale images is possible in the above-mentioned platform. The results show high variability of the annotation speed between readers, which may help determine annotator engagement in the process. Demirer et al. (14) showed a locally designed graphical user interface, where a single radiologist performed single-label classification and object detection of proximal femoral fractures on radiographs. Expert annotators spent about 10 sec per study (IQR: interquartile range, 3-21 sec per study), labeling over 1,000 radiographs over 7 days. Their study results confirm that a layout familiar to radiologists might speed up the annotation process. However, their solution was limited in terms of scalability and accessibility outside of a single institution.

This research study presents the development of a zero-footprint, web-based tool that is easy to implement on both the local and global scale. Our approach suggests using a minimal number of features for the user-friendliness of the interface. Furthermore, we provide annotators with additional clinical information, including radiological reports or patient history, to improve annotation accuracy as an option. The results of high-reliability annotation can be obtained by providing annotators with information comparable with the real clinical environment. To combat the widely known problem of ‘soft ground truth’, imaging does not always correlate with hidden clinical information.

Previously, a few other image annotation tools were presented in the literature. Rubin et al. presented *ePAD*, an online platform for quantitative imaging (15). Their solution was focused more on clinical trials and cancer imaging, providing annotation templates for quantitative image features and tools for size measurements and several additional plugins. The proposed platform did not have tools or workflows optimized specifically for tasks typical in computer vision. *LesionTracker* is a platform with very similar functionalities, which is also dedicated to cancer imaging research (16).

Other developed tools, such as *RIL-Contour* proposed by Philbrick et al., are often more task specific (17). The authors presented software focused on volumetric annotation, especially image segmentation. They also included using locally developed AI models and displaying saliency maps to understand model interference better. However, their solution is not web-based and synchronized, which drastically limits the potential for crowdsourcing annotations. In addition, they used the NifTI file format instead of the DICOM Standard. *DeepLNAnno* proposed by Chen et al., which is another web-based system that implements deep learning models inside the platform for pre-annotation (18). Having said that their solution is explicitly dedicated to lung nodule annotation in CT studies. In contrast, *MarkIt* attempts to overcome previous research limitations, presenting a robust platform dedicated to CNN-based ML or AI research in the medical imaging field. Connecting multiple RESTful services allows this platform to scale and rapidly increase further functionality.

The blockchain technology has been widely recognized to deliver decentralization and transparency to solutions in many areas. Some attempts have been made in medicine to utilize those benefits, mostly when handling electronic health records, promising better management for data ownership, sharing, or authorization (19). Nevertheless, only rarely attempts to utilize blockchain have resulted in developing a tool useful in the clinical setting. Our experiments explored how the blockchain technology could encourage transparency and trust when crowdsourcing annotations are practiced by saving their activity in an immutable ledger. The blockchain technology has the advantage of defending against data manipulation without the installation of an additional security system. We could achieve security for image upload or annotation record modulation without compromising user convenience. In the future, blockchain might give incentives for annotators or inspire anonymous data sharing. It is still not fully clear when and where the blockchain technology might serve better than the standard approaches (e.g. traditional databases) in radiological tools, but further research will most likely maximize their benefits.

Currently, *MarkIt* still has a few limitations. First, our software does not yet include tools for image segmentation. Although Cornerstone.js library does offer basic segmentation tools, we believe that other tools, such as 3D Slicer, are more appropriate for complex image segmentation, which often requires more complex workflow and multiple manual and/or semi-automatic segmentation processes. Second, at present, our platform does not fully support volumetric images and non-image DICOM instances, such as DICOM-SEG and DICOM-SR. Finally, although *MarkIt* enables users to upload DICOM and non-DICOM (e.g. JPG) image files, several other simple imaging formats are commonly used in the radiology research community, for example, NIfTI (especially in the neuroimaging community) or NRRD (Nearly Raw Raster Data). As this study was conducted by three radiologists of similar levels belonging to a hospital, it would be exciting to see how annotators of various hospitals’ at different levels participated.

Although not yet realized, the blockchain technology will enable us to obtain a free and fair data exchange system among researchers in the near future. Currently, most of the data are owned by healthcare providers, major national research institutes, and large research institutes. Ultimately, sharing all data without condition will help create a new value. Still, it would not be easy to actively share data without compensation for intellectual labor, such as annotation and curation, reflecting the benefits of the institution that owns it.

We aim to continue to improve the *MarkIt* system and leverage the potential of blockchain technology to reflect the value of the data and use it as a currency for data transactions in the near future. The issuance of currencies with a specific purpose for data exchange may also help in data acquisition for AI development while being less likely to cause ethical problems related to data ownership issues.

## Conclusions

The value of data is variable, and therefore, it would be challenging to adjust prices according to supply and demand principles. Our mathematical evaluation tool is based on various factors, and it reflects the accuracy of annotators, the balance of labels, and the depth of information. Researchers can quickly evaluate the value of a dataset and prevent data contamination caused by the wrong annotation.

We used the mean annotation time to measure each label’’s labor intensity, and the AI prediction value was used to estimate the ground truth. Owing to the nature of Cloudsourcing notations, it is difficult to clearly assess the ability of annotators, leading to problems in estimating the ground truth by majority rule. The participation of many people who do not have prior knowledge of the annotation task can cause serious problems with data reliability.

It will be an exciting research topic on how *Markit* and Blockchain technologies can induce positive effects on crowdsourcing annotation and data exchanges. We presented a collaborative annotation platform dedicated to medical imaging. The *MarkIt* system efficiently performs crowdsourcing annotation and provides indicators to evaluate the value of data and efforts of annotators.

## Supplementary Material

*MarkIt:* A Collaborative Artificial Intelligence Annotation Platform Leveraging Blockchain For Medical Imaging Research
