# Supplementary material for: MarkIt: A Collaborative Artificial Intelligence Annotation Platform Leveraging Blockchain For Medical Imaging Research
Source: Blockchain Healthc Today. 2021 Jun 22;4:10.30953/bhty.v4.176. doi: 10.30953/bhty.v4.176 (PMC9907418; doi:10.30953/bhty.v4.176)
Supplement: MarkIt: A Collaborative Artificial Intelligence Annotation Platform Leveraging Blockchain For Medical Imaging Research [file BHTY-4-176-s001.pdf]

Supplementary Material 1. List of classification labels used in the chest X-ray annotation proof of concept project.

1. Foreign body/Lines and Tubes
2. Foreign body/External
3. Foreign body/Hardware
4. Foreign body/Other
5. Lung volume - Decreased
6. Lung volume - Increased
7. Lung density - Increased - Pneumonia like lesions
8. Lung density - Increased - Atelectasis
9. Lung density - Increased - Pulmonary edema
10. Lung density - Increased - Interstitial lung disease
11. Lung density - Decreased - Emphysema
12. Lung density - Decreased - Cavity/Cyst
13. Hilar/mediastinum - Mediastinum
14. Hilar/mediastinum - Aorta
15. Hilar/mediastinum - Cardiomegaly
16. Pleura - Pneumothorax
17. Pleura - Other pleural lesions
18. Diaphragm
19. Abdomen/Free air
20. Bone - Fracture
21. Bone - Spinal deformity
22. Lung density - Increased - Nodule/mass
23. Hilar/mediastinum - Hilar area
24. Pleura - Pleural effusion
25. Unacceptable image

Supplementary Material 2. Mean duration for annotation.

Label	95% confidence interval of mean duration (seconds)
Pneumonia	5.83 ± 0.79
Atelectasis	6.05 ± 0.58
Pleural effusion	5.43 ± 0.55
Cardiomegaly	4.63 ± 0.54
Pulmonary edema	7.27 ± 1.28
Interstitial lung disease	10.79 ± 4.53
Pneumothorax	13.92 ± 3.93

Supplementary Material 3. Cryptocurrency shares for each annotators.

Label	Annotator A	Annotator B	Annotator C	Total
Pneumonia	141	147	113	401
Atelectasis	233	258	234	725
Pleural effusion	146	119	143	408
Cardiomegaly	159	122	58	339
Pulmonary edema	66	43	17	126
Interstitial lung disease	13	14	15	42
Pneumothorax	10	15	4	29
Total share	768	718	584	2070
(Cryptocurrency)	(371T)	(346T)	(282T)	(1000T)

Supplementary Material 4. Cohen’s kappa between AI and annotators.

Label	Cohen’s kappa		
	AI - Annotator A	AI - Annotator B	AI - Annotator C
Pneumonia	0.444	0.430	0.459
Atelectasis	0.673	0.645	0.659
Pleural effusion	0.808	0.778	0.794
Cardiomegaly	0.563	0.655	0.555
Pulmonary edema	0.551	0.644	0.323
Interstitial lung disease	0.150	0.167	0.183
Pneumothorax	0.240	0.202	0.071
